# Concept Design for a 1-Lead Wearable/Implantable ECG Front-End: Power Management

**DOI:** 10.3390/s151129297

**Published:** 2015-11-19

**Authors:** Libin George, Gaetano Dario Gargiulo, Torsten Lehmann, Tara Julia Hamilton

**Affiliations:** 1 School of Electrical Engineering and Telecommunications, University of New South Wales, Sydney 2052, Australia; E-Mail: t.lehmann@unsw.edu.au; 2 The MARCS Institute, Western Sydney University, Penrith 2751, Australia; E-Mails: g.gargiulo@westernsydney.edu.au (G.D.G.); t.hamilton@westernsydney.edu.au (T.J.H.)

**Keywords:** DC-DC, reconfigurable, wearable, ECG, sensor

## Abstract

Power supply quality and stability are critical for wearable and implantable biomedical applications. For this reason we have designed a reconfigurable switched-capacitor DC-DC converter that, aside from having an extremely small footprint (with an active on-chip area of only 0.04 mm${}^{2}$), uses a novel output voltage control method based upon a combination of adaptive gain and discrete frequency scaling control schemes. This novel DC-DC converter achieves a measured output voltage range of 1.0 to 2.2 V with power delivery up to 7.5 mW with 75% efficiency. In this paper, we present the use of this converter as a power supply for a concept design of a wearable (15 mm × 15 mm) 1-lead ECG front-end sensor device that simultaneously harvests power and communicates with external receivers when exposed to a suitable RF field. Due to voltage range limitations of the fabrication process of the current prototype chip, we focus our analysis solely on the power supply of the ECG front-end whose design is also detailed in this paper. Measurement results show not just that the power supplied is regulated, clean and does not infringe upon the ECG bandwidth, but that there is negligible difference between signals acquired using standard linear power-supplies and when the power is regulated by our power management chip.

## 1. Introduction

As the global population of persons over 60 years of age continues to increase, health-care solutions that reduce the burden on an already stressed health-care and welfare system are required. As a result, there is growing interest in the development of technologies that can enable health-care services to be administered in the comfort of patients’ homes [1,2]. One such technology is the monitoring of bio-signals, particularly electrocardiograms (ECGs). The relatively wide availability of microelectronics with embedded software, open access wireless protocols and longer battery life has led to the research and development of wearable, wireless bio-sensor systems that can be worn on the body and integrated into clothing.

It is important for such devices to have a stable and effective power supply accompanied by a good power management system in order to maximize battery life and thus avoiding the need for replacement batteries or frequent recharging. Simultaneously, these devices must be small and light-weight to fit in the paradigm of wearable/implantable devices. Additionally, such devices need to be affordable to enable wide adoption. For these reasons, fully integrated low-cost power management solutions are becoming imperative [3,4].

Switched-capacitor (SC) DC-DC power converters are a suitable choice for these devices [5,6]. They require less die area overhead and are therefore low-cost and more compact than standard switched-mode power supplies. With new technologies, switching at high frequencies with less power loss has become attainable and the area overhead can be further reduced [7]. SC converters are a good option for biomedical devices as they can be switched at high frequencies without compromising on efficiency; thus they can be small in size and give less electromagnetic interference (EMI) than inductor-based designs [8,9]. As well as optimising the size and efficiency of the power supply, we must also deal with varying load and voltage requirements to facilitate adaptive medical devices with various modes of operation: sleep, measurement, stimulation, transmission of data, *etc*. SC converters with reconfigurable power stages can eliminate the need for re-design, reduce area and provide a wide range of output voltages to cater for these different operations.

In this paper, we propose the concept design of a wearable ECG front-end device with particular focus on the design of a novel power regulation and management chip. This chip will be used in the proposed system to regulate power to an ECG front-end circuit and, in future iterations of the design, a microcontroller from an unregulated source such as an energy-harvester. The power management chip is small and the converter architecture and control methods have features that are advantageous to the operation of such a wearable biomedical device. Test measurements have been taken to verify the operation of the ECG front-end with the power management chip and results show that it works extremely well in this application, making it possible to measure ECG signals that are accurate and free of additive distortion.

## 2. Background

The treatment of many illnesses can be better managed by monitoring patients for extended periods of time while they perform their normal daily activities. Such forms of patient monitoring require equipment to satisfy specific requirements such as portability and/or wearability, low power consumption, durable electrodes, data security and compliance with medical device regulations such as electrical safety, electromagnetic compatibility and so on [10].

One branch of medical science that could certainly benefit from long-term monitoring is cardiology. Morphological changes or the presence of various arrhythmias in ECG are known to have a strong correlation with heart and coronary artery diseases [11]. Apart from being useful to follow up on patients whose medical condition is already known, long-term ECG monitoring can also be used to monitor athletes during exercise. Sudden cardiac emergencies, sometimes even death, continue to occur amongst professional athletes [12]. “Athlete’s heart” syndrome can be associated with several alterations and abnormalities present in the ECG [13]. It is widely accepted that the origin of the ECG alteration cannot be reliably clarified using the standard 12-lead ambulatory ECG [14]. A system that is able to record the ECG during exercise accurately and without interference is highly desirable.

Standard ECG measurements are taken using electrodes attached to the patient’s skin. The skin is first prepared by cleaning, shaving, abrasion to remove dead skin and moistening. A layer of electrically conductive gel is then applied to the skin area where electrodes are then attached. The gel is used to reduce contact impedance [15]. However, the quality of the recorded signals from these “wet electrodes” declines over time and the gel dehydrates. Also, the gel can occasionally leak and can lead to electrically shorting the recording sites on the skin. Securing these electrodes in place is also quite complicated as the electrodes cannot be directly glued to the skin. Therefore, the use of dry or insulating electrodes can avoid or reduce these problems [16].

Many research groups are now developing wearable, wireless bio-sensor systems for applications such as health-care and monitoring of athletes and vital signs in high-risk environments. Whilst some systems focus on particular applications usually measuring only one or two signals [17], other groups are looking into network methods for body sensor networks (BSNs), body area networks (BANs) and personal area networks (PANs) [18]. The aim here is to develop miniature, low-power nodes which are worn on the body. Each node is capable of sampling, processing and communicating physiological measurements. In wireless networks, the nodes need to wirelessly communicate to a central hub, eliminating the need for cables between sensors. This improves long-term monitoring by making it more comfortable and practical, but does raise new challenges such as power, size and security requirements on the nodes.

Radio-frequency identification (RFID) enabled sensors provide effective solutions for wireless BANs and other biomedical applications [19]. RFID tags contain an integrated circuit and antennae which are used to transmit to a RFID reader. RFID can be used to transmit ECG data that has been measured to the user and thus facilitates monitoring of the patient in real-time, avoiding the need for large data storage on the device itself.

The RFID sensor and the ECG front-end on such a device require a good power source along with appropriate power regulation and management. Battery-powered sensors are common in many wearable applications but are not necessarily ideal. They add size and weight and require frequent recharging or replacement, making them less desirable. Energy-harvesters, on the other hand, can help overcome the trade-offs between battery volume, battery lifetime and system functionality in wearable bio-sensors [20]. They provide an inexhaustible source of energy and also provide user convenience, a fairly important factor in the design of such devices. Energy must then be used effectively for all necessary functions (e.g. ECG measurement, data processing, RF transmission). Power regulation and distribution must be both stable and efficient: stability to ensure that no vital measurements are lost, measured and processed data are not corrupted, and high efficiency to maximise the usage of the energy being harvested. The power supply must also deal with any changes in the source’s input voltage seamlessly.

During the last few years researchers and manufacturers have proposed a number of different solutions for smart power supply management (particularly power scavenging) systems that address the needs of power efficient biomedical devices. As a result, along with several proposed experimental/research devices (see [6,21,22] in Table 1), there are a plethora of off-the-shelf solutions available on the market. Unfortunately, the vast majority of these solutions are integrated power modules which, despite not requiring external components (use of small decoupling capacitors is often recommended), are big, bulky and often have an unregulated output and strict constraints for the input voltages. This is because these devices also provide galvanic insulation and ground loop elimination. For applications where galvanic insulation is not required, there are several relatively small (SOIC packages) SC integrated solutions which are commercially offered (see [8,9] in Table 1). Despite being relatively small these solutions may still require an inductor and in most cases they require at least four external capacitors (10 μF or larger) to work.

**Table 1:** Experimental/Research DC-DC Converters for Sensor Applications.

Features	[6]	[21]	[22]	[8]	[9]
Active Die Area	0.52 mm${}^{2}$	0.57 mm${}^{2}$	4.56 mm${}^{2}$	N/A	N/A
On-chip Capacitor	300 pF	2.4 nF	6.72 nF	N/A	N/A
Off-chip Devices	0	0	0	8	2+
No. of Conversion Ratios	3	5	1	2	1
Operation	Step-down	Step-down	Step-down	Step-down	N/A
Input Voltage, V${}_{IN}$	1.2 V	1.2 V, 1.8 V	2.5 V	3.3 V or 5 V	5 V, 15 V or 24 V
Output Voltage, V${}_{OUT}$	0.3–1.1 V	0.3–1.1 V	0.8–1.5 V	3.3 V or 5 V	5 V, 15 V or 24 V
Output Power	1–230 μW	5 μ–1 mW	0.4–7.5 mW	<400 mW	1 W
Efficiency Range	30%–80%	50%–80%	50%–66.7%	34%	<85%

The lack of a truly attractive power management solution, particularly from those solutions available commercially, has lead us to develop the SC power converter that we present in this paper. Firstly, our system must occupy a small device area and volume: reducing the size of the power solution is key to the development of a smaller and lighter wearable application. Current solutions such as shown in Table 1 occupy much larger die areas due to the use of large integrated pump capacitors. Off-chip components must be reduced or eliminated to further reduce total device size and weight, and also improve mechanical reliability of the application; a fully-integrated power solution is advantageous to this cause. Depending on the energy-harvesting options available, a solution providing both step-up and step-down DC-DC conversion is useful in providing supply voltages that are lower and higher than the voltage range catered by the harvesters. Most existing solutions, such as those shown in Table 1, provide step-down conversion only and are not optimal under conditions where the energy harvested is insufficient to maintain the required voltage whilst driving the same output load. In such circumstances, step-up voltage conversion becomes useful. A wide output voltage range is necessary for the wearable device and decent efficiency must be maintained in its various modes of operation. Unlike existing converters that are either small but cater for smaller power output [6], or can deliver much larger power but occupy much larger die area [22], a small, high power solution is very desirable for our proposed wearable application. Finally, a reconfigurable SC converter architecture is required in the power management solution to provide multiple conversion ratios and be able to deal with any changes to either the input voltage sourced by an energy-harvester and/or the output voltage required for a different mode of system operation (such as sleep, ECG measurement, data transmission). Such a converter architecture will allow re-use of the SC DC-DC converter to serve a wide range of voltages and loads, reducing design time and reducing total area [23,24]. The design solution presented in this paper presents such a reconfigurable SC converter suitable for a wearable ECG front-end application that is small, cost-effective and power efficient, using a novel system architecture and modern control schemes.

## 3. System Architecture

**Figure 1:**
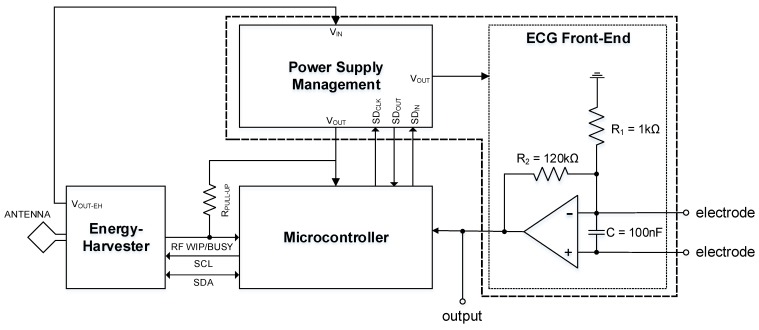
System architecture of the proposed concept prototype. The focus of this paper is the design and evaluation of the power supply module and the ECG front-end circuit.

Figure 1 shows the system architecture of the 1-lead wearable ECG front-end device that we propose for our application. It consists of an energy-harvester chip, a power management chip, a microcontroller and ECG front-end circuitry. The energy harvester scavenges RF energy using its antenna. This energy is stored and delivered to the power supply management integrated circuit (IC) as an unregulated voltage, $V_{OUT-EH}$. In turn, the power management chip uses the energy sourced by the harvester to provide a regulated power supply, $V_{OUT}$, to the rest of the system comprising of the microcontroller and the ECG front-end. ECG measurements are taken using two electrodes and the measured analogue ECG signal is delivered to the microcontroller which digitises and stores the data before transmitting the data to the user via the RF channel provided by the energy-harvester chip. The dimension of this proposed sensor system is dictated by the antenna size (approximately 15 mm × 15 mm [25]).

As can be inferred from Figure 1, the power supply is the most important block in the system. The stability of the power supply is critical to the functioning of the microcontroller and to the quality of the ECG signal measured. Therefore, there is a need for a stable power supply compatible with the power scavenging requirement. We have focused our efforts in the design and testing of a custom solution that can be used in this system.

The following sub-sections briefly describe the ECG front-end, the energy-harvesting chip and control circuits. The main focus of this paper, the power management chip, is then detailed in Section 4. Finally, test results showing successful operation of the power management chip with the ECG front-end circuit are presented in Section 5.

### 3.1. ECG Front-End Interface

The ECG front-end interface consists of an operational amplifier, two resistors, a capacitor and two electrodes, as shown in Figure 1. For the purposes of our tests, we devised the values of $R_{1}$, $R_{2}$ and *C* to implement a bandwidth of 0.05 Hz–550 Hz and gain of 120 to allow correct acquisition of data by the data acquisition device. Our circuit bandwidth adheres to diagnostic ECG requirements [2]. A TI OPA2333 chip containing two micropower CMOS operational amplifiers was used in the test prototype. The single-lead ECG design that we propose uses only one of these op-amps and consumes a maximum of 25 μA quiescent current. This chip is small, consumes very little power and is readily available, making it ideal for our application. Also, it can operate at a 1.8 V supply voltage, as provided by the power management chip. The same chip can also be used in a 2-lead ECG front-end application.

### 3.2. Energy Harvesting, Control Signals and Data Processing

For the sake of completeness, we briefly describe the remaining parts of our proposed sensor system. The STMicroelectronics^®^ M24LR16E-R chip (Geneva, Switzerland) is an RFID-tag IC with 16-kbit EEPROM memory, energy-harvesting, I^2^C and RF interfaces. This chip is ideal for our application. The energy harvesting mode at a field strength of 1.0 A/m can deliver a minimum 18 mW of power and a voltage output range of 2.7–4.5 V. This suits our power supply chip which can step-down the input voltage to a regulated output voltage $V_{OUT}$ of 1.8 V for the other modules in the system. The memory and I^2^C interface are useful for the communication and storage of digitised ECG data received from the microcontroller chip before being transmitted to the user over the RF channel. The multiple password protection schemes available in its RF mode provide added security and make it even more suitable for multi-sensor network applications in which our system can be used.

An ultra-low power microcontroller chip by STMicroelectronics^®^, STM32L476xx, is used as the controller to the system. One of the PLLs available is used to clock serial data transmission between the power management and microcontroller chips ($SD_{CLK}$). Control data is sent to the power management chip serially ($SD_{IN}$) and feedback data ($SD_{OUT}$) monitoring the performance of the voltage regulator can be returned to the microcontroller for system analysis and recording, if required. The embedded ADC on this chip is used to digitise the ECG data received from the ECG front-end module and the digitised information is then transmitted via the RF channel in the energy-harvester chip. This chip also operates at the 1.8 V supplied by the power management chip.

## 4. Power Supply Management

The power supply and management chip has to meet several challenges. Firstly, it must provide a clean supply to the ECG front-end circuitry. It is important that the voltage ripple (caused by the regulator’s switching) is outside the bandwidth of ECG measurements. The chip must also provide a reliable supply to the microcontroller in the system to ensure that its on-chip memory is not corrupted and ECG data is processed and transmitted to the RF device correctly. Finally, it is vital that the power management chip can regulate voltages for variable loads: output current load requirements vary between different modes of operation of the other modules, such as sleep, ECG measurement, ECG data processing and data transmission. Voltage regulation is also required during current spike events that may occur when the amplifier in the ECG front-end circuit saturates: this is a common occurrence with biopotential amplifiers where saturation occurs as a response to artifacts brought about by movements and large EMI in the environment.

**Figure 2:**
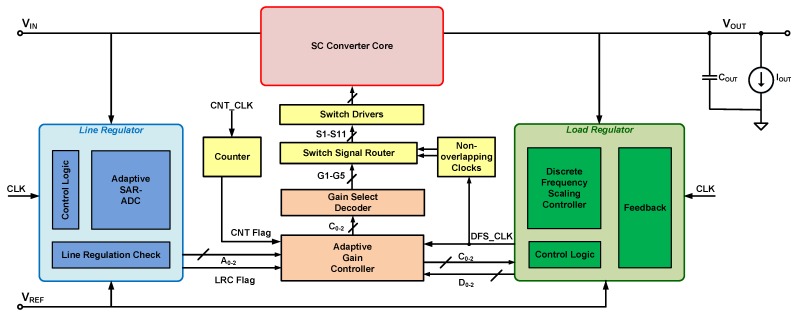
Architecture of the reconfigurable SC DC-DC converter system [5,27].

Figure 2 shows the top-level architecture of the reconfigurable SC power converter that we propose to use in the system for power regulation and management to the microcontroller and ECG front-end circuit. In this prototype converter, a 5-gain ratio (GR) power stage (based on [26]) was used. This SC core circuit uses 11 power transistor switches and 2 pump capacitors to achieve 5 different GRs, performing both buck (step-down) and boost (step-up) operations. The multiple GRs available in this reconfigurable SC architecture (1/2, 2/3, 1, 3/2 and 2) make it possible for the converter to adapt to any variation in the unregulated supply from the energy-harvester and provide the necessary power required to maintain proper operation of the ECG front-end and faultless data processing and memory operations in the microcontroller. This is especially important during high current consuming modes such as during system start-up, flash memory write operations and data transmission by the RFID chip, and ECG amplifier saturation.

An adaptive SAR-ADC is used to determine the required GR from the input voltage, $V_{IN}$, and the target output voltage, $V_{REF}$. The load regulator indicates the location of the converter’s output voltage, $V_{OUT}$ with respect to $V_{REF}$. An adaptive gain controller (AGC) uses the information provided by the line and load regulators to control the appropriate GR of switching required in the SC converter power stage using two non-overlapping clock phases, $\phi_{1}$ and $\phi_{2}$. The AGC ensures that changes in the supply voltage or the load are quickly identified and the optimum current is always supplied, providing efficient operation of the power management chip. A switch signal router circuit routes the required switching signals to switch driver circuits that turn on the corresponding power transistors in the switched-capacitor power stage and thereby provide the load current, $I_{OUT}$, which is described by

(1) $$I_{OUT}=I_{ECG}+I_{\mu C}$$

where $I_{ECG}$ is the current consumed by the ECG front-end circuit and $I_{\mu C}$ is the current consumed by the microcontroller.

Once $V_{OUT}$ is within a region close to $V_{REF}$, the adaptive gain control is stopped and a discrete frequency scaling controller (DFSC) adjusts the switching frequency, $f_{S}$, in discrete steps to fine-tune $V_{OUT}$ towards $V_{REF}$. Also, once $V_{OUT}$ reaches $V_{REF}$, the switching of the converter core is disabled and pulse-skipping (*i.e.*, allowing $V_{OUT}$ to drift downwards and reactivating switching when it drops below $V_{REF}$ again) is enabled to keep the output regulated. The use of pulse-skipping significantly improves power consumption by reducing switching losses. This is an important consideration in this application which must make efficient use of the energy available from the harvester.

**Figure 3:**
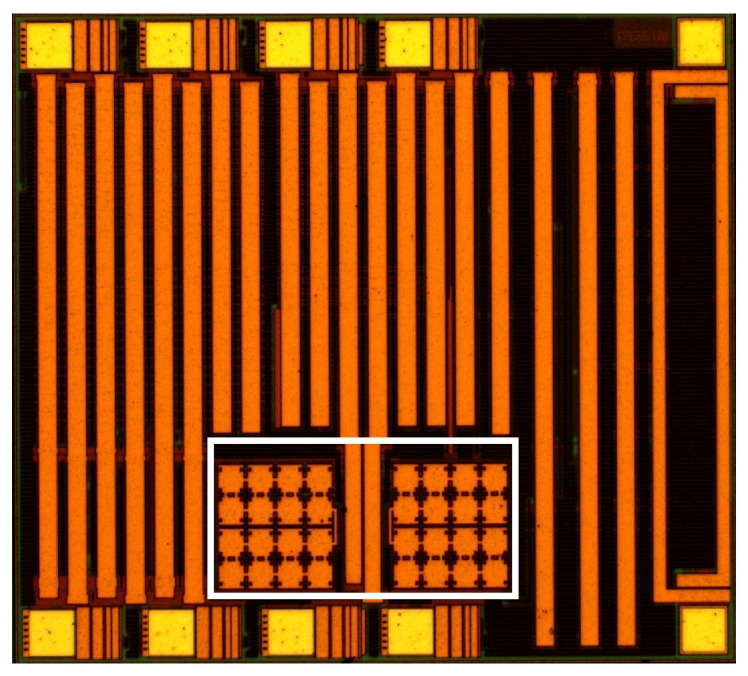
Chip microphotograph.

The reconfigurable SC DC-DC converter incorporating all these design features (detailed in [5]) was implemented and fabricated in a 0.18 μm standard bulk CMOS n-well process. The converter was designed to be a fully integrated solution, excluding an output filter capacitor, $C_{OUT}$, which may be required as a separate off-chip component depending on the capacitance of the load. In this design, we have balanced the flexibility required by our system with efficiency and power consumption; a trade-off was necessary. Figure 3 shows the die microphotograph of the power management chip. The chip has a size of 890 μm × 870 μm. The converter occupies the area of 400 μm × 200 μm, within the marked box. Active circuitry of the SC converter system consumes just 0.04 mm^2^. High density (4 fF/mm2) Metal-Insulator-Metal (MIM) capacitors available in the technology were used to occupy minimal area and they also allowed all the control circuitry and core power transistors to be laid underneath their respective layers, thus further reducing the total area occupied by this fully integrated solution. The two charge pump capacitors $C_{p1}$ and $C_{p2}$ in the SC converter power stage were sized to be 80 pF each due to external constraints on the die area that was available. Given the size of these capacitors and the off-chip filter capacitance, a suitable switching frequency, CLK, was chosen to keep the $V_{OUT}$ ripple small (<1% of $V_{OUT}$). The maximum clock frequency of switching employed in the design was 20 MHz. The key specifications of the power converter are shown in Table 2. The converter can easily cater the size of load currents required by the system and for all modes of operation in the microcontroller as shown in Table 3.

The SilTerra CL180G process technology that was used in the design of this prototype chip provides both 1.8 V and 3.3 V devices, but only 1.8 V devices were used in this iteration. For the design of the proposed system, the next iteration of the chip will use the 3.3 V devices instead. The use of the higher voltage devices will allow the design of an on-chip band-gap reference that can be used to set a reference voltage $V_{REF}$ of 1.8 V required for the ECG front-end circuit. This reference voltage could be designed to be programmed by the microcontroller to facilitate different modes of operation such as sleep, record, transmit, *etc*. making the design highly flexible and ensuring minimum power consumption. The chip will be sourced by the 2.7–4.5 V supplied by the energy-harvester and shall provide a regulated step-down voltage of 1.8 V to the ECG front-end sub-system. The switching clock, CLK, will be provided by an on-chip clock generator.

**Table 2:** Key features of the DC-DC converter.

Technology	0.18 μm bulk CMOS
Active Die Area	0.04 mm^2^
On-chip Pump Capacitance	2 × 80 pF
Type of On-chip Capacitance	Dual-MIM
Number of conversion ratios	5
Operation	Step-up & Step-down
Input voltage, $V_{IN}$	1.8 V
Output voltage, $V_{OUT}$	1.0–2.2 V
Output power	100 μ–7.5 mW
Efficiency range	30%–75%
Figure of Merit (*m*W/mm^2^)	750
Operating frequency	1.25/2.5/5/10/20 MHz

**Table 3:** Current consumption of the microcontroller during various modes of operation.

Mode	Description	Current Consumption ^1^
Run	Normal operation	100 μA/MHz
Sleep	CPU Disabled	35 μA/MHz
Shutdown	Device turned OFF	0.33 μA with RTC^2^

^1^ Typical consumption at $V_{DD}$ = 1.8 V, 25 °C, Flash memory off;

^2^ Real-time clock enabled.

## 5. Results and Discussion

### 5.1. General Power Supply Performance

**Figure 4:**
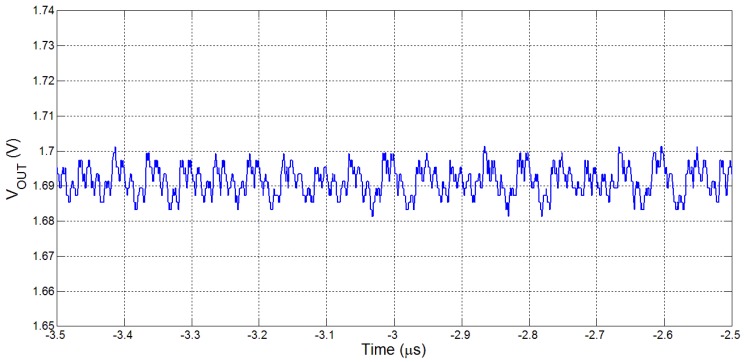
Measured $V_{OUT}$ regulation at 1.70 V for a load current of 300 μA.

**Figure 5:**
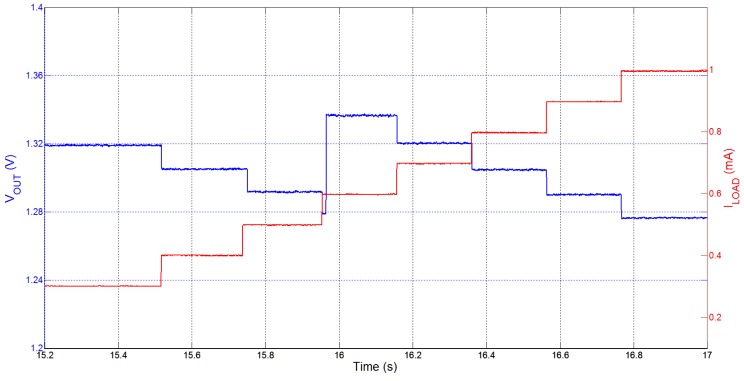
Measured $V_{OUT}$ regulation at 1.30 V as the load current steps from 300 μA to 1 mA in steps of 100 μA.

The performance of the power management chip was evaluated using a general but extensive bench test for a wide range of output voltages and load currents. Here, we present some key results showing important features of our power converter that are very useful to the system that we have proposed. Firstly, Figure 4 shows $V_{OUT}$ regulation at 1.70 V and a ripple voltage of less than 20 mV for a load current of 300 μA. Figure 5 is a measured plot showing load regulation of $V_{OUT}$ at 1.30 V and it shows how the output voltage is regulated as the load current varies, such as could happen during different modes of operation of the microcontroller. In this example, the load current is incremented from 300 μA to 1 mA, in steps of 100 μA. In this scenario, the steady-state $V_{OUT}$ level drops by approximately 13 mV with every increment of the output current. This drop in $V_{OUT}$ is a function of both the change in output current, $\Delta I_{OUT}$, and the output resistance of the SC circuit for the gain ratio at which the converter operates at the given time. The power converter uses both the adaptive gain control and the discrete frequency scaling control to ensure that $V_{OUT}$ does not deviate too far from the target (in this case, 1.3 V) at a load of 600 μA. If changes in switching frequency (handled by the DFSC) are insufficient to maintain fine-regulation of $V_{OUT}$ to within ±2%–3%, the AGC generates a step increment in GR to enable more power to be delivered and thereby return $V_{OUT}$ to regulation. Such a feedback loop reduces the impact of any change in load current. For future iterations of this power converter, size optimization of the power transistors in the SC circuit can be used to improve the effective output resistance for each gain ratio used in the system. Further work has already been done since this iteration to provide finer control in load regulation using a novel DFS scheme [28].

**Figure 6:**
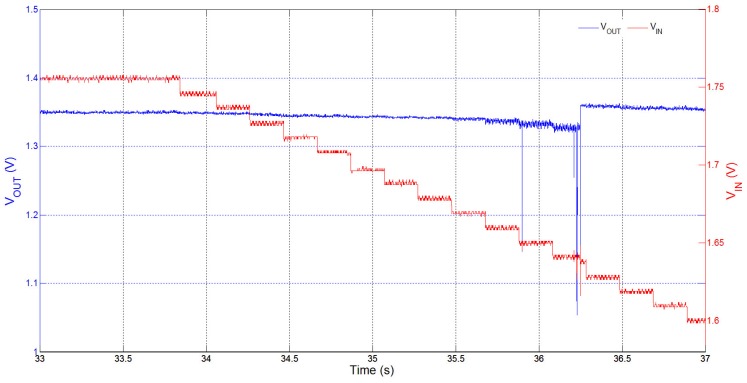
Measured $V_{OUT}$ regulation at 1.35 V as the supply voltage, $V_{IN}$, drops from approximately 1.76 V to 1.60 V in steps of 10 mV.

In Figure 6, we show the response of the power converter to a variation in the supply voltage $V_{IN}$. In this example, we see how $V_{OUT}$ is regulated while $V_{IN}$ decreases from approximately 1.76 V to 1.60 V in steps of 10 mV. As expected from the design features of the converter, the change in supply voltage initially does not affect the regulation of $V_{OUT}$. However, when $V_{OUT}$ eventually begins to drop, and $V_{OUT}$ can no longer be regulated at $V_{REF}$ by the current gain ratio and the maximum frequency of switching by DFS control, the AGC makes a change in gain ratio to increase the power being delivered and raise $V_{OUT}$ back into regulation. Such a feature in the reconfigurable power converter is very advantageous to our power supply solution as it can ensure that the microcontroller and ECG front-end circuitry are unaffected even under circumstances when there is a drop in the voltage supplied by the energy-harvester.

**Figure 7:**
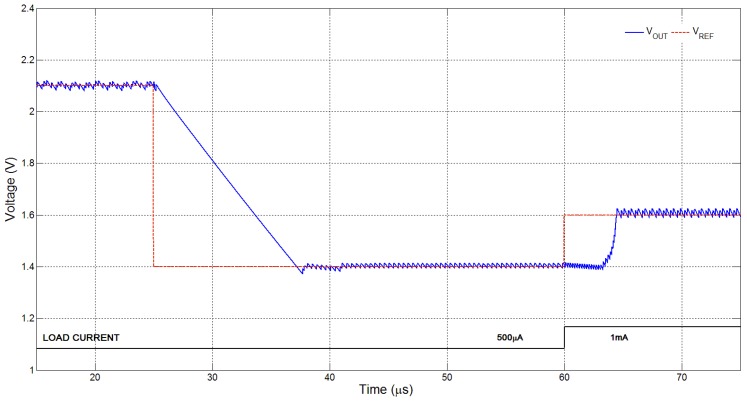
Simulation plot showing an example of voltage tracking.

**Figure 8:**
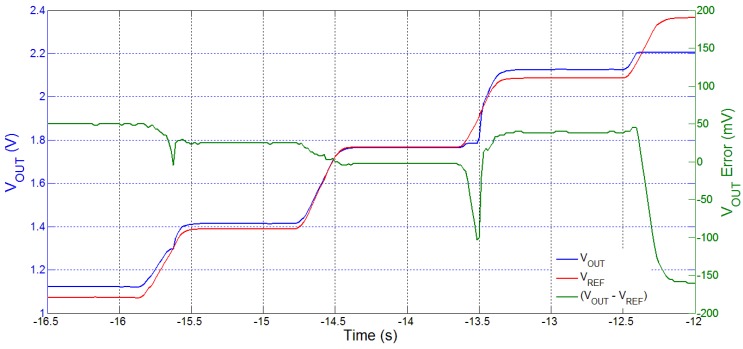
Measurement plot showing voltage tracking of $V_{REF}$ for a load of 10 μA.

Figure 7 shows a simulation example of voltage tracking and regulation for changing $V_{REF}$ and load current, $I_{OUT}$. As seen in the figure, the converter adapts to maintain regulation of $V_{OUT}$ at different target voltages ($V_{REF}$) within the voltage and power range. Such voltage tracking is very useful to our proposed system: the AG, DFS and pulse-skipping schemes features in the power management chip ensure that load regulation can be achieved and maintained with dynamic changes in voltage and current output requirements, such as those that occur during changes in operation mode. Figure 8 is a measurement plot showing how $V_{OUT}$ varies as $V_{REF}$ is swept over a wide range. The steady-state $V_{OUT}$ error over this range can be observed to be within ±50 mV for most of the operating $V_{OUT}$ range.

### 5.2. ECG Front-End with Power Supply Results

ECG contains important information in the bandwidth of 0.5 Hz–150 Hz [2]. It is therefore important that the power management chip is not only capable of driving the front-end circuit but also does not corrupt the measured ECG data by the addition of any harmonics into the output signal. A very-low power ECG front-end device was realised using the circuit described in Section 3.1. The measured bandwidth of our setup is 0.05 Hz–550 Hz, which works well for our ECG application. The suitability of our power management chip to provide the ECG power supply was then tested and verified by a frequency analysis of the measured ECG data. For the purpose of this experiment, we recorded ECG data at a very high sample rate (48 kHz) using a National Instruments Data Acquisition (DAQ) board (NI6009). The ECG signals were acquired from a Medsim 300b patient simulator using a standard calibrated 1 mVpp Lead-I at the standard rate of 60 beats per minute (bpm). More than 30 min of ECG data (from a single lead) were recorded: firstly using a direct power supply of 1.8 V obtained by a standard lab power supply, and secondly from a 1.8 V supplied by our chip from a 1.5 V coin-cell battery used as a test energy source. The coin-cell battery was used because it provides a clean energy source, making it possible to determine the additive noise and distortion that is contributed by the power management circuit. We challenged the performance of the power management chip by comparing the regulated 1.8 V output voltage it provides to that from an Agilent B2912A Precision Source/Measure Unit (SMU) power supply.

**Figure 9:**
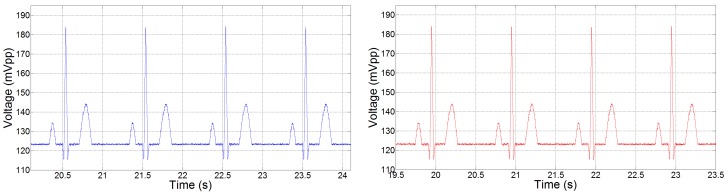
Measured ECG readings (scaled by amplifier gain) when using a direct power supply of 1.8 V (shown on **left**) and a regulated 1.8 V from the power management chip (shown on **right**) using a 1 mVpp Lead-I at 60 bpm.

**Figure 10:**
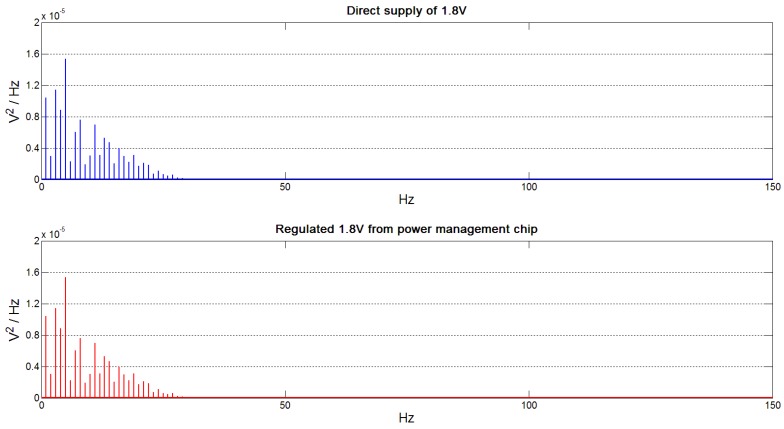
FFT plots of the ECG measurements taken when the ECG front-end circuit is powered by: (**i**) a direct external 1.8 V power supply; and (**ii**) a regulated 1.8 V from the integrated DC-DC converter discussed in Section 4.

Figure 9 shows excerpts of ECG data recorded from the ECG front-end circuit. It shows that ECG readings have been successfully and correctly measured. The ECG readings that were obtained by powering the ECG front-end with the lab SMU, and then by the power management chip, were found to be nearly indistinguishable. Figure 10 shows a plot of FFT analysis of ECG data measured from the direct power supply and that of ECG data measured from the regulated supply from the power management chip. As shown by the plots, there is little to no difference between the frequency components of both supplies, proving that the prototype power management DC-DC converter that we propose works very well for our application. These figures demonstrate that we can obtain a clean ECG signal from the ECG front-end circuit and that the power management chip does not introduce any unwanted frequencies in the ECG bandwidth. Figure 9 and Figure 10 also show that our power management chip compares extremely well with standard lab power supplies and can be used to provide good voltage regulation from a battery source. In order to quantify the near-identical FFT plots (Figure 10), we report the amplitude of the frequency components in Table 4.

**Table 4:** FFT peak magnitudes of ECG when ECG front-end circuit is powered using: (i) a direct external 1.8 V power supply; and (ii) a regulated 1.8 V from the integrated DC-DC converter discussed in Section 4.

Frequency (Hz)	Magnitude (mV^2^/Hz)		Frequency (Hz)	Magnitude (mV^2^/Hz)	
	(i)	(ii)		(i)	(ii)
1	10.4234	10.4226	11	6.9753	6.9474
2	2.9902	3.0083	12	3.0970	3.0818
3	11.3771	11.3936	13	5.2705	5.2627
4	8.8463	8.8393	14	4.6910	4.6708
5	15.3526	15.3551	15	2.0290	2.0366
6	2.2612	2.2434	16	3.9635	3.9561
7	6.0397	6.0470	17	2.9964	2.9826
8	7.5853	7.5680	18	2.2474	2.2485
9	1.8924	1.8835	19	3.0910	3.0777
10	3.0389	3.0401	20	1.7296	1.7179

**Figure 11:**
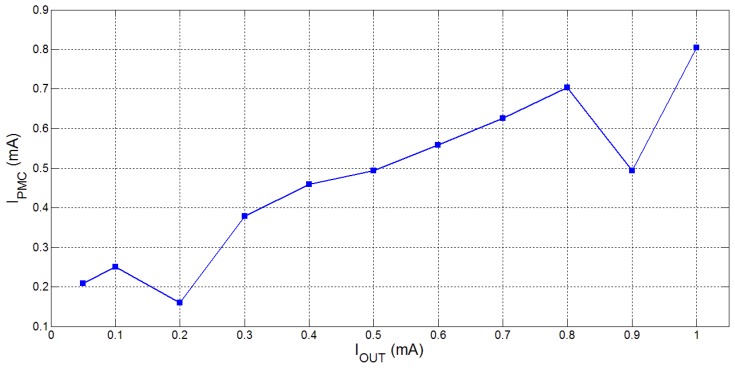
Power consumption of the power management chip for different load currents while regulating the output voltage, $V_{OUT}$, at 1.8 V.

The current consumed by the power management chip, $I_{PMC}$, depends on the output voltage, $V_{OUT}$, and the load current, $I_{OUT}$, being driven, as is therefore a function of the gain ratio (set by the AGC) and the switching frequency (set by the DFSC). In Table 5, we show the current consumption of the SC converter system while powering the ECG front-end circuit with a 1.8 V supply. The quiescent current consumption of the SC converter can range between 0.2 to 0.8 mA depending on the load, as shown in Figure 11; this is acceptable for our intended application and within the current amount that can be supplied by the M24LR16E-R chip energy-harvester chip. Some minor changes have also been made to the SC converter system to further reduce static power consumption and these changes have already been implemented for the future prototype [5,28].

We have shown the successful operation of a power management chip with an ECG front-end circuit. The power management chip consists of a novel reconfigurable SC converter that uses adaptive gain and discrete frequency scaling control techniques to provide a regulated 1.8 V to the ECG front-end circuit. However, in our test setup, the power management chip was sourced by a 1.5 V coin-cell battery, and hence required a step-up DC-DC conversion to the voltage required by the operational amplifier in the ECG front-end circuit. The next steps in the design of the proposed 1-lead wearable ECG front-end device will combine the use of the power management and ECG modules with the microcontroller and energy-harvester chips described in Section 3.2. The ADC in the microcontroller will be used to digitise the ECG signal and store the information in memory. At fixed time-intervals, the microcontroller will transmit the stored data along with timing information to the energy-harvester chip over I^2^C communication, which in turn transmits the data to the user over the RF channel available on the chip.

**Table 5:** Measured current consumption of the power management and ECG front-end circuits when supplying a regulated output voltage of 1.8 V.

	Circuit	Current Consumption
1	ECG Front-end Circuit Only (Source: 1.8 V Power Supply)	27.2741 μA
2	Power Management Chip with ECG Front-end Circuit (Source: 1.5 V Battery)	0.2577 mA

Apart from the extension of the system to include the other modules, the power management chip also requires some design modifications and improvements.

### 5.3. Device Type and Source Voltage

The use of higher voltage CMOS devices will allow the use of the higher source voltage that will be provided by the energy-harvester than that which was feasible in this early prototype. The SC converter can perform a step-down conversion of the voltage from the harvester to the supply voltage required by the microcontroller and ECG front-end circuit.

### 5.4. On-Chip Reference Voltages

Unlike this iteration of the power management chip that uses an external voltage reference to provide the target voltage for SC converter output, the next chip will contain on-chip reference voltages. Operating voltages greater than the maximum of 1.8 V that was possible in this prototype can be achieved by the use of the higher voltage CMOS devices and the design of an appropriate band-gap reference.

### 5.5. Clock Generator

An on-chip clock generator such as in [29] will be added to the power management chip: this is important to ensure that self-start of the power management chip is possible at system start-up. Once the power management chip has started its operation and $V_{OUT}$ regulation is achieved, the microcontroller and ECG front-end circuits will begin working and ECG measurements, processing, recording and transmission will then be enabled by their respective circuits.

### 5.6. Multiple Outputs

This reconfigurable SC power converter can be extended to provide multiple regulated outputs [28], which can be used to power the digital and analogue supplies of the microcontroller separately.

## 6. Conclusions

In this paper, we have proposed a feasible system architecture of a wearable ECG-front device that uses an energy-harvester and a power converter to supply regulated power to an ECG front-end circuit that can measure ECG signals and a microcontroller that can be used to process and transmit the ECG data to the user. We also presented the novel power management chip that can be used to supply and maintain the power required to the proposed system. This chip contains a reconfigurable SC converter that has design features and specifications that are very well suited to its application within this wearable ECG front-end system. Tests were conducted to verify the quality of the regulated power supply provided by this chip to the ECG front-end circuit, and results have shown that the power supply is clean and free of interference within the standard ECG bandwidth. Future work will see the development of an upgraded power management chip and its integration with an energy-harvester and a microcontroller to complete the proposed wearable system and enable the measuring of ECG signals and their transmission to the end-user.
